# A role for a *Trypanosoma brucei* cytosine RNA methyltransferase homolog in ribosomal RNA processing

**DOI:** 10.1371/journal.pone.0298521

**Published:** 2024-04-25

**Authors:** Kevin T. Militello, Jennifer Leigh, Matthew Pusateri, Laurie K. Read, Dineen Vogler

**Affiliations:** 1 Biology Department, State University of New York at Geneseo, Geneseo, NY, United States of America; 2 Department of Microbiology and Immunology, Jacobs School of Medicine and Biomedical Sciences, Buffalo, NY, United States of America; University of Toronto, CANADA

## Abstract

In *Trypanosoma brucei*, gene expression is primarily regulated posttranscriptionally making RNA metabolism critical. *T*. *brucei* has an epitranscriptome containing modified RNA bases. Yet, the identity of the enzymes catalyzing modified RNA base addition and the functions of the enzymes and modifications remain unclear. Homology searches indicate the presence of numerous *T*. *brucei* cytosine RNA methyltransferase homologs. One such homolog, TbNop2 was studied in detail. TbNop2 contains the six highly conserved motifs found in cytosine RNA methyltransferases and is evolutionarily related to the Nop2 protein family required for rRNA modification and processing. RNAi experiments targeting TbNop2 resulted in reduced levels of TbNop2 RNA and protein, and a cessation of parasite growth. Next generation sequencing of bisulfite-treated RNA (BS-seq) detected the presence of two methylation sites in the large rRNA; yet TbNop2 RNAi did not result in a significant reduction of methylation. However, TbNop2 RNAi resulted in the retention of 28S internal transcribed spacer RNAs, indicating a role for TbNop2 in rRNA processing.

## Introduction

*Trypanosoma brucei* is the causative agent of African Sleeping Sickness and nagana in cattle and wildlife. The life cycle of the parasite is complex, as it necessitates both insect and mammalian hosts and requires fundamental changes in metabolism and surface protein expression [1]. Not surprisingly, changes in gene expression accompany the complex life cycle changes. In *T*. *brucei*, transcription occurs for clusters of genes present on the same strand which are called polycistronic transcription units [2]. Transcriptional regulation for polycistronic transcription units is apparently absent [2]. Thus, processes such as mRNA trans-splicing, coupled cleavage and polyadenylation, RNA editing of mitochondrial mRNAs, and regulation of RNA stability and translatability have been heavily investigated. Although the abundance, processing, and translational efficiency of *T*. *brucei* RNAs have been extensively analyzed by sequencing analysis [3], less is known about the chemical modifications of *T*. *brucei* RNA that impact RNA metabolism.

RNA sugars and bases can be chemically modified to generate a complex pool of RNA whose totality is called the epitranscriptome [4–7]. The presence of modified bases in tRNA has been known for many years. To date, there have been greater than 100 modified bases identified in tRNA including N^1^-methyladenosine, dihydrouridine and 5-methylcytosine (m^5^C) impacting several processes including codon recognition, tRNA charging and reading frame maintenance [8–10]. Similarly, rRNA is extensively modified, and the modifications can impact RNA processing, ribosomal assembly, and/or organismal growth [11]. Although it was clear from past work that tRNA and rRNA are modified, the field has been moved by the discovery of N^6^-methyladenosine demethylase FTO [12], and subsequent identification of N^6^-methyladenosine in the mRNA body and cap, where the presence of the modified base impacts virtually all aspects of mRNA metabolism [13].

One modified base that has generated a lot of attention is m^5^C, as it has been heavily studied as an epigenetic mark in DNA controlling several processes including transcription and gene silencing [14]. Yet, m^5^C is found in several RNA species as well [15]. Eukaryotic tRNAs contain multiple m^5^Cs. Hotspots for m^5^C in eukaryotic tRNAs include the anticodon loop, the anticodon stem, the variable region, and the acceptor stem [15]. m^5^C in tRNA has been linked to numerous processes such as blocking rapid tRNA decay [16], protection against stress induced tRNA cleavage [17], improved stability/translatability of specific codons [18], and control of innate immunity [19]. Based on studies in yeast and humans, 28S eukaryotic rRNA is methylated at two unique positions; one is present at the interface between the small and large ribosomal subunits and the other site is near the peptidyl transfer center [15]. The presence of m^5^C in rRNA has been linked to changes in antibiotic sensitivity, ribosomal RNA processing, ribosomal protein composition, and ribosomal assembly [11,20–22]. The discovery of the presence of m^5^C in non-coding RNA stimulated the search for m^5^C in mRNA. The presence of m^5^C in eukaryotic mRNA has been reported several times as well [23–25], and it is clustered in the 5’ and 3’ UTRs. Although a defined function for m^5^C in mRNA is less clear, it may impact nuclear export and translation [23,25]. Other RNA species such as long non-coding RNAs and vault RNAs contain m^5^C as well [15].

The enzymes that methylate RNA at cytosine residues are called CRMTs for cytosine RNA methyltransferases [26]. CRMTs transfer the methyl group from S-adenosylmethionine to position 5 of the pyrimidine ring. CRMTs share numerous motifs with DNA methyltransferases including motifs necessary for S-adenosylmethionine (SAM) binding, cytosine recognition, and catalysis [27,28]. CRMTs contain two conserved cysteine residues that are necessary for the initial attack of the cytosine ring and release from the cytosine after methylation [29,30]. Cytosine RNA methyltransferase enzymes have been best characterized from baker’s yeast. ScTrm4 methylates tRNA [31], while ScNop2 and ScRcm1 methylate the two aforementioned rRNA sites [20,22,32]. Interestingly, ScNop2 is also required for rRNA processing, but it is unclear if this is related to its methylation activity or is a separate function [22,32–34]. Human cells contain homologs of all the yeast enzymes; these enzymes are referred to as NSUNs (Nol1/Nop2/Sun RNA methyltransferase). Human cells contain additional NSUNs compared to yeast presumably due to extra potential RNA targets not present and/or not methylated in yeast including vaults RNAs, mitochondrial rRNA, and mRNAs [15,26].

In *T*. *brucei*, there have been several RNA modifications identified that impact RNA metabolism, indicating *T*. *brucei* does contain a complex epitranscriptome [35]. Examples of important RNA modifications include base and sugar methylation of the mRNA cap4 structure [36], spliced leader RNA pseudouridylation [37], rRNA sugar methylation [38], rRNA pseudouridylation [39] and adenosine methylation of VSG mRNA poly(A) tails [40]. Yet, we are just beginning to scratch the surface on identifying modified cytosine bases in RNA [35]. Rubio *et al*. reported that a 3-methylcytosine residue was present in tRNA, and was required for RNA editing via deamination [41]. With respect to m^5^C, our group reported on the presence and location of m^5^C in *T*. *brucei* tRNA using sodium bisulfite sequencing [42]. In this work, we found m^5^C in the variable region at C48-C50 but not at C38 in the anticodon or anticodon loop. The function of m^5^C in *T*. *brucei* tRNA is currently unknown. In addition, it is currently unknown if other *T*. *brucei* RNA species are modified by m^5^C addition and the consequences of the modifications. Therefore, in this report, we searched for the presence of CRMT enzymes and their potential targets as a strategy to uncover the function of m^5^C in *T*. *brucei* RNA.

## Materials and methods

### Bioinformatic analyses

**Fig 1:**
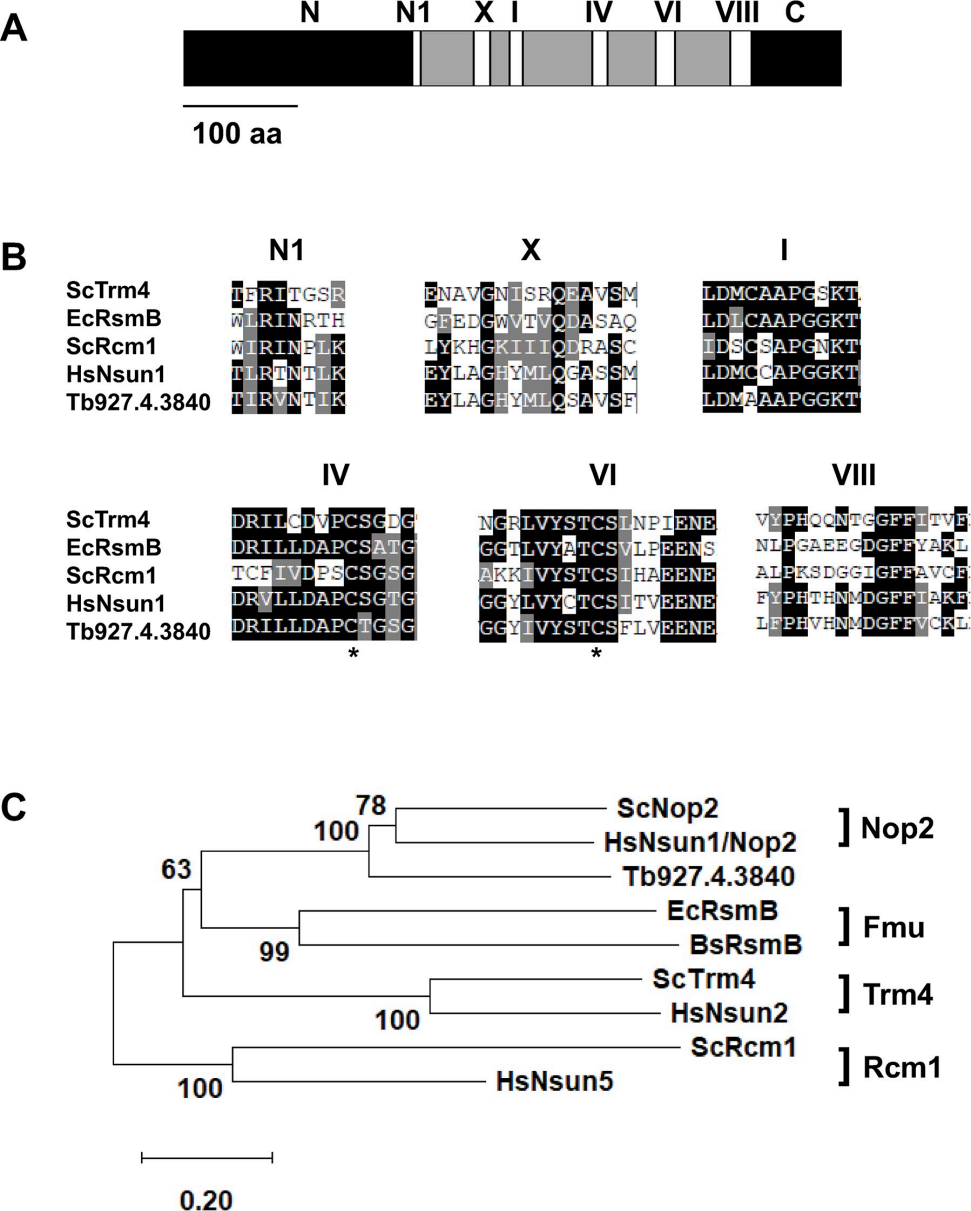
Structure and phylogeny of TbNop2. A) Domain structure of Tb.927.4.3840 (TbNop2). N is the N-terminal region, C is the C-terminal region, and Roman numerals represent six highly conserved motifs found in other cytosine RNA methyltransferases and DNA methyltransferases. The numbers are based on cytosine DNA methyltransferase domains. B) Alignment of Tb927.4.3840 (TbNop2) to experimentally validated cytosine RNA methyltransferases. Identical amino acids are shaded black and structurally conserved amino acids are shaded grey (>50% prevalence). Only the six most highly conserved motifs in A are shown. Asterisks represent the two catalytic cysteines in motifs IV and VI. C) Unrooted neighbor joining tree including Tb927.4.3840 (TbNop2) and members of the four main cytosine RNA methyltransferase subfamilies (Nop2, Fmu, Trm4, and Rcm1). Bootstrapping values are indicated (1000 bootstraps). Proteins included in the analysis include BsRsmB (*Bacillus subtilis*, P94464), EcRsmB (*Escherichia coli*, P36929), HsNsun1/Nop2 (*Homo sapiens*, P46087), HsNsun2 (*Homo sapiens*, Q08J23), HsNsun5 (*Homo sapiens*, Q96P11), ScNop2 (*Saccharomyces cerevisiae*, SGD:S000005005), ScTrm4 (*Saccharomyces cerevisiae*, SGD:S000000120), and ScRcm1 *(Saccharomyces cerevisiae*, SGD:S000004967). All identification numbers are from UniProt except for *Saccharomyces cerevisiae* proteins (yeastgenome.org).

BLASTP searches of *T*. *brucei* TREU 927 predicted proteins present in TriTrypDB were carried about using standard conditions (E-value cut off E<1x10^-5^) [43]. Protein alignments were generated with T-coffee [44], and modified using BoxShade. For phylogenic analysis, Mega 11 software was used [45]. Protein sequences were aligned with MUSCLE. Unrooted, neighbor-joining trees were generated; 1000 bootstraps were performed. The partial deletion setting for gaps was used with a cut-off of 80% site coverage. All alignment and tree member accession numbers are provided in the Fig 1 legend.

### *Trypanosoma brucei* culture and strain construction

*T*. *brucei* strain 29–13 and its derivates below were used [46], and cultured in SM media containing 10% fetal bovine serum at 27°C with 15 μg/mL G418 and 50 μg/ml hygromycin to maintain selection of the T7 RNA polymerase and tetracycline repressor genes [47]. To create a C-terminally Ty-tagged version of TbNop2, strain 29–13 was transfected with a Ty-tagging cassette obtained by PCR amplification of the pPOTv4-Ty-Blastocidin vector with primers corresponding to the 3’ end of the TbNop2 ORF without the stop codon and the 5’ end of the 3’UTR of TbNop2 [48]. See S1 Fig for primer sequences. Resulting TbNop2-Ty cells were selected with 20 μg/ml blasticidin and cloned by limiting dilution. To create an inducible RNAi system against TbNop2, a 906 basepair fragment of the TbNop2 gene was amplified by PCR (see S1 Fig for primer sequences), purified with a Qiagen PCR purification kit, digested with XbaI and HindIII and inserted into the XbaI/HindIII sites of plasmid p2T7-177 to create pTbNop2RNAi [49]. Not1-lineared pTbNop2RNAi was introduced into *T*. *brucei* 29–13 cells containing Ty-tagged TbNop2 by electroporation [50] and selected using 2.5 μg/ml phleomycin. Cells were cloned by limiting dilution. For growth curve analyses, parasite cultures were grown in the absence and presence of 4 μg/mL doxycycline for 10 days at 27°C. Parasites were enumerated with a hemocytometer (n = 3).

### TbNop2 expression

TbNop2 was detected in parasite extracts via immunoblotting. A mouse anti-TY antibody was generated at the University of Alabama Monoclonal Antibody Facility. The secondary antibody was an HRP-conjugated goat anti-mouse antibody (Sigma). The *T*. *brucei* p22 protein was detected as a loading control [51]. Original blotting images are provided in S2 Fig.

### Total RNA isolation and qRT-PCR

Total RNA was isolated using Trizol. Total RNA (~0.5–1 μg) was treated with DNA-free DNase (Life Technologies) and purified using either a Qiagen RNeasy column or Zymo Research RNA Clean and Concentration column. All RNAs were analyzed by Bioanalysis on an RNA 6000 Nano Chip at the University of Rochester Genomics Research Center (Rochester, NY). RNA was converted to cDNA using the New England Biolabs Protoscript kit using random primers. qPCR was performed on the cDNA in reaction volumes of 20–30 μL. Each reaction contained 1x Sybr green master mix (Bio-Rad), 0.1 μM forward primer, 0.1 μM reverse primer, and cDNA template. Primer sequences are provided in S1 Fig. Reaction conditions were 95°C for 10 minutes, 95°C for 30 seconds, 60°C for 1 minute, 72°C for 1 minute for 40 cycles. Melting curves were performed by heating samples from 65°C to 95°C for 30 minutes. Fold-change calculations were performed using the ΔΔCt method using β-tubulin for normalization [52]. Experiments were performed 4 times (n = 4). One-sample t-tests were utilized to determine the significance of the qRT-PCR fold changes. Fold-change values for experimental genes were tested against the value of 1, which represents no change (RNAi-on/RNAi-off).

### BS-seq (Bisulfite sequencing)

Two μg of DNase-treated total RNA from two separate cultures of TbNop2-Ty RNAi cells grown in the absence and presence of 4 μg/mL doxycycline for 2 days was spiked with 0.2 ng of luciferase RNA (Promega), and treated with 4 cycles of sodium bisulfite using the Qiagen EpiTect Bisulfite Kit as previously described [42,53,54]. TruSeq stranded libraries were created at the University of Rochester Genomics Research Center (Rochester, NY). Oligo(dT) selection was not used in order to maintain high rRNA levels. Limited RNA fragmentation conditions were used as bisulfite treatment cleaves RNA. Bisulfite-treated RNAs were analyzed by next generation sequencing on a NovaSeq 6000 machine at the University of Rochester Genomics Research Center (Rochester, NY). Single-end sequencing was used for 75 cycles. The sequence files are present at the Sequence Read Archive (https://submit.ncbi.nlm.nih.gov/subs/sra/) within BioProject ID PRJNA996772. Sequences were analyzed using Galaxy [55]. Adapters were removed using Trim Galore, and sequences were reverse complemented. Mapping and methylation calls were performed using Bismark and MethylExtractor [56]. Reads were mapped to the firefly luciferase coding mRNA sequence lacking the poly(A) tail to determine the deamination rate. Reads were mapped to a set of chromosome 3 *T*. *brucei* TREU 927 rRNAs (Tb927.3.3421-Tb927.3.3428) to assess rRNA methylation (S3 Fig). Internal transcribed spacers were included as separate “genes”. Cytosines with less than 10-fold coverage and cytosines from potential antisense transcription events were removed from further analysis [57]. A stringent cut-off was used to call m^5^Cs. Cytosines were called m^5^C if they displayed >50% methylation in both RNAi-off samples, and were surrounded by adjacent cytosines with <50% methylation in both RNAi-off samples to avoid regions with poor deamination rates [58]. Two sample T-tests were used to compare methylation levels between RNAi-off and RNAi-on samples. All statistical tests were performed in R [59].

### BS-seq confirmation experiments

For RNA methylation confirmation experiments, the Zymo Research EZ RNA methylation kit was used according to the manufacturer’s instructions. 1 μg of total RNA was treated with sodium bisulfite. Approximately 10 ng of bisulfite-treated RNA was converted to cDNA using the New England Biolabs Protoscript Kit. Random primers were used. Specific rRNA fragments were amplified by PCR (see S1 Fig for primer sequences). Primers were designed in regions outside of the putative methylation sites and their design assumed complete deamination in these regions. PCR conditions were 94°C for 2 minutes, 40 cycles of 94°C for 1 minute, 50°C for 1 minute, and 72°C for 1 minute, followed by 72°C for 10 minutes. PCR products were cloned into the pGEM-T Easy plasmid and introduced into *Escherichia coli* JM109. Plasmids were isolated from single colonies and sequenced at Genewiz (now Aventa technologies). 10 or more plasmids were sequenced from each experiment.

## Results

### Cytosine RNA methyltransferase identification

To identify *T*. *brucei* putative enzymes that methylate RNA, the well characterized *Saccharomyces cerevisiae* Trm4 tRNA methyltransferase protein sequence was used in BLAST searches of *T*. *brucei* TREU 927 predicted proteins [31]. Seven hits were obtained with E-values <1E^-^5. These molecules were initially named CRMT 1–7 for cytosine RNA methyltransferases 1–7 (S1 Table). This indicates that *T*. *brucei* has multiple proteins with the potential to methylate RNA. As a first step to examine the significance of the different CRMTs, we analyzed the previously published RNAi target sequencing (RIT-seq) data. In RIT-seq, RNAi libraries targeting protein coding genes were grown under non-inducing conditions (RNAi-off) and inducing conditions (RNAi-on). Genomic DNA was isolated after RNAi-induction and analyzed by next generation sequencing to identify underrepresented genomic fragments, suggesting a loss of fitness associated with silencing the target gene with RNAi [60]. We were drawn to the fifth homolog identified in our bioinformatic searches, Tb927.4.3840, as Tb927.4.3840 was underrepresented in both procyclic form and bloodstream form RNAi library experiments in RNAi-on samples. Tb927.4.3840 is the primary focus of this article.

The predicted Tb927.4.3840 protein is 65 kDa in size. The Tb927.4.3840 protein sequence was aligned to known, experimentally validated CRMTs to determine if it contains the six highly conserved motifs found in cytosine RNA methyltransferases [27,28]. These motifs are also found in cytosine DNA methyltransferases. These motifs have functions in SAM binding, attack of pyridine ring, and release of the enzyme from the pyrimidine ring. Indeed, Tb927.4.3840 has the six highly conserved motifs found in DNA and RNA methyltransferases including the cysteines necessary for the initial attack on the cytosine ring in motif VI and release from the cytosine ring in motif IV (Fig 1A and 1B). This suggests that Tb927.4.3840 has the potential to methylate nucleic acid targets. There are four main subfamilies of CRMTs [27]. To determine the relationship between Tb927.4.3840 and CRMT subfamilies, phylogenic analysis was used. Tb927.4.3840 is most highly related to the Nop2 subfamily containing the *S*. *cerevisiae* Nop2 protein (ScNop2) and the human Nsun1 protein (HsNsun1) (Fig 1C). Both ScNop2 and HsNsun1 are rRNA methyltransferases that also function in rRNA processing and ribosome assembly [22,32–34,61]. Thus, for simplicity, Tb927.4.3840 was referred to as TbNop2.

### TbNop2 tagging and RNAi

**Fig 2:**
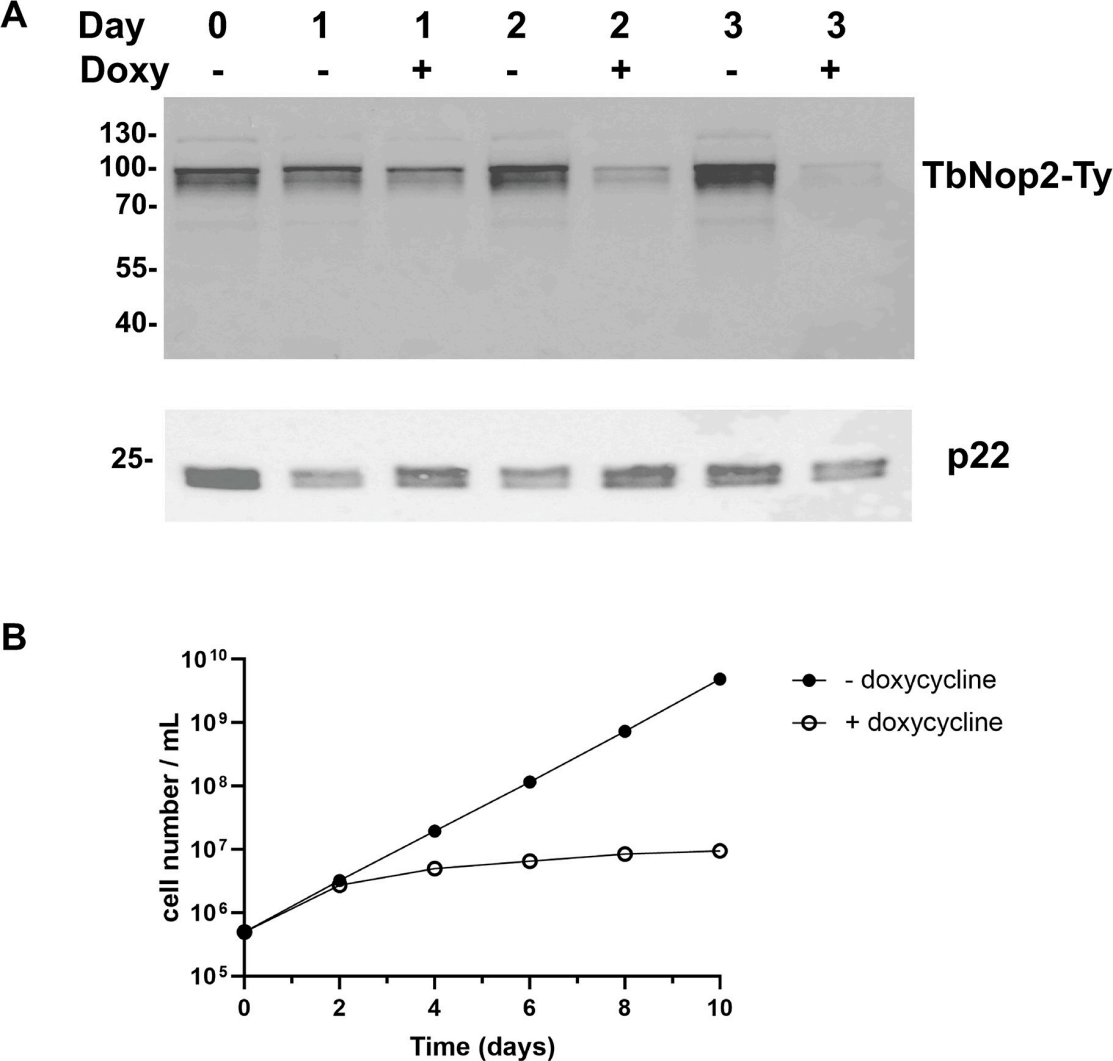
TbNop2 tagging and targeting with RNAi. A) Procyclic form parasites carrying an endogenous Ty-tagged TbNop2 gene were engineered and modified by the introduction of a doxycycline-inducible TbNop2 RNAi system. TbNop2-Ty RNAi cells were grown in the absence and presence of 4 μg/mL doxycycline for 3 days. Protein extracts were separated by SDS-PAGE on days 1–3, an analyzed via immunoblotting with an anti-Ty antibody (top). The membrane was also probed with an antibody against the *T*. *brucei* p22 protein as a loading control (bottom). A representative experiment is shown. B) TbNop2-Ty RNAi cells were grown in the absence of doxycycline (RNAi-off) and split into two separate flasks. Doxycycline was added to the second flask at a final concentration of 4 μg/mL (RNAi-on). Cell cultures were grown for ten days (and subcultured when necessary). Every two days, parasites were enumerated using a hemocytometer and split accordingly. Each curve is the average of three separate experiments (n = 3). Error bars represent standard deviation, and may be smaller than the symbols used.

To track the presence of TbNop2, a Ty-tagged version of TbNop2 was created and expressed from the endogenous locus in procyclic form parasites. A protein of the expected size of the Ty-tagged protein (75kDa) was detected in total cell extracts via western blot analysis (Fig 2A). To investigate the importance of TbNop2, RNAi was utilized. Procyclic form TbNop2-Ty parasites expressing TbNop2 double-stranded RNA were created, and double-stranded RNA was induced by the addition of doxycycline. The addition of doxycycline for 1–3 days clearly reduced the levels of the Ty-tagged TbNop2 protein (Fig 2A). qRT-PCR was also utilized to examine the effect of TbNop2 RNAi on native TbNop2 RNA levels. TbNop2 RNA levels were reduced 5.7-fold in the presence of doxycycline for 2 days (p = 2.2x10^-5^). Overall, the results indicate that targeting TbNop2 with RNAi reduces TbNop2 RNA and protein levels. Experiments measuring parasite growth were performed by growing TbNop2-Ty RNAi parasites in the absence (RNAi-off) and presence of doxycycline (RNAi-on) for ten days (Fig 2B). In the presence of doxycycline, TbNop2-Ty RNAi cells lost the ability to divide after two days, suggesting an important function(s) for TbNop2. The data are consistent with the aforementioned RIT-seq data [60].

### rRNA methylation in TbNop2 RNAi parasites

TbNop2 shares the six highly conserved motifs found in DNA and RNA cytosine methyltransferases including the cysteines present in motifs IV and VI (Fig 1A and 1B). TbNop2 is also closely related to the Nop2 protein family that methylates the large rRNA (Fig 1C). In addition, previous localization studies indicated that TbNop2 resides in the nucleolus [62]. Thus, we hypothesized that TbNop2 methylates rRNA as does the yeast ScNop2 and human HsNsun1 proteins. To determine if *T*. *brucei* rRNA contains m^5^C and if TbNop2 methylates rRNAs *in vivo*, a sodium bisulfite sequencing strategy was utilized. Total RNA was isolated from TbNop2-Ty RNAi cells grown in the absence (RNAi-off) and presence of doxycycline for two days (RNAi-on). Total RNA was spiked with a small amount of luciferase RNA that is not methylated to measure deamination efficiency. Total RNA was treated with sodium bisulfite and used for library preparation in the absence to oligo(dT) selection to maintain high rRNA levels. Bisulfite-treated RNA was then analyzed by next generating sequencing. Sequencing reads were mapped to the firefly luciferase gene and *T*. *brucei* TREU 927 rRNA cluster including the internal transcribed spacer (ITS) sequences to identify the position of remaining cytosines (5-methycytosines or m^5^Cs). Using this approach, Cs are deaminated to Us and read as Ts during DNA sequencing. Methylated Cs are not deaminated and are read as Cs during DNA sequencing. This approach was pioneered for DNA methylation studies [63], but has been converted to RNA methylation [53,54].

First, the firefly luciferase RNA data were analyzed. The firefly luciferase RNA, which is not methylated, was an important negative control for the experiment. The average percentage methylation across four samples was 3.9%, indicating the deamination rate is 96.1% (Table 1). These findings indicate that the deamination conditions for the experiment are robust resulting in efficient deamination of non-methylated cytosines. Next, the TbNop2-Ty RNAi samples were analyzed from samples grown in the absence of doxycycline (RNAi-off). 1704 total cytosines with greater than 10-fold coverage were analyzed from the rRNA cluster (S2 Table). Signals for most rRNA species including the ITSs were present and robust (~500,000X coverage) (Table 1 and S2 Table). However, little coverage was observed for M6 rRNA and ITS4, possibly due to their small size. Only two cytosines showed strong evidence for the presence of methylation (defined as methylation level >50%, see Materials and Methods). These were sites 541 and 1324 in 28Sβ rRNA (Table 1). These sites are homologous to the two methylated sites in baker’s yeast and humans highlighting the conservation of these two rRNA sites in eukaryotes [22,64]. Importantly, the homologous site to 1324 is methylated by Nop2 proteins in yeast and human rRNAs.

**Table 1:** Bisulfite sequencing analysis of *T*. *brucei* rRNA.

Luciferase deamination rate	96.1%
rRNA genes/ITSs analyzed	15
Cytosines analyzed	1704
average fold coverage^a^	571,909
cytosines >50% methylation^a^^,^^b^	2
methylated targets	28Sβ rRNA positions 541 (98.3%) and 1324 (99.0%)

^a^ based on TbNop2 RNAi off samples.

^b^ see Materials and Methods for criteria.

**Fig 3:**
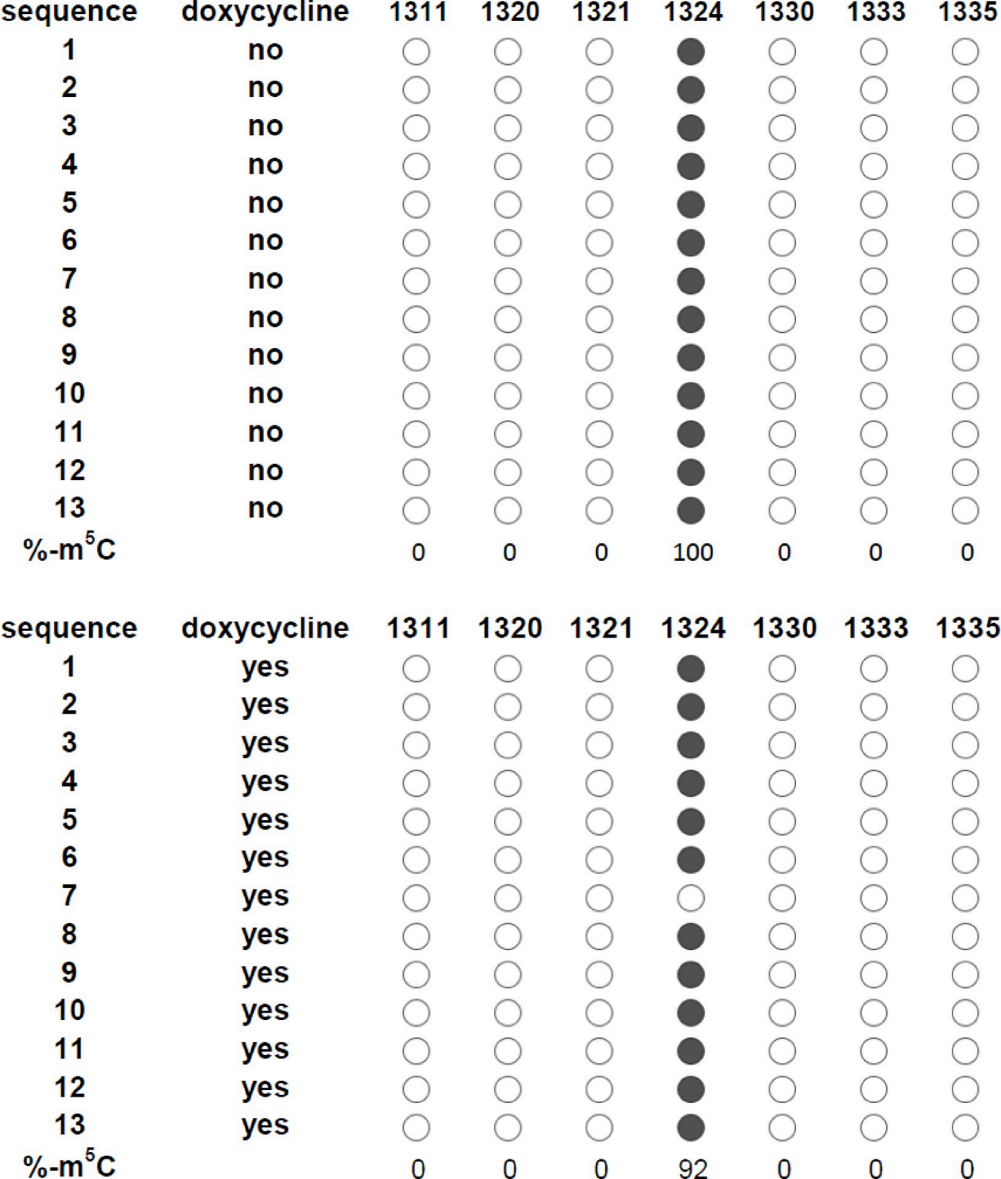
Sodium bisulfite sequencing analysis of 28Sβ rRNA site 1324. Total RNA was isolated from TbNop2-Ty RNAi cells grown in the absence and presence of doxycycline for 2 days. RNA was treated with sodium bisulfite (converting non-methylcytosines to uracils) and converted to cDNA. A small 28Sβ rRNA region containing position 1324 was amplified by PCR. Products were inserted into pGEM-T Easy plasmids and analyzed by Sanger sequencing (13 plasmids per sample). The region containing site 1324 and three surrounding cytosines are shown: m^5^Cs are indicated with filled circles, where non-m^5^Cs are indicated with open circles.

We next analyzed the bisulfite sequencing dataset to determine if methylation of 28Sβ rRNA sites 541 and/or 1324 was reduced in the presence of TbNop2 RNAi. The level of 28Sβ rRNA cytosine methylation at position 541 was unchanged in the presence of TbNop2 RNAi (0% reduction). The level of 28β rRNA cytosine methylation at position 1324 was slightly reduced by 2.9% by TbNop2 RNAi. T-test analysis indicated the change in methylation at site 1324 was not statistically significant (p = 0.47). To further evaluate the BS-seq data, we analyzed site 1324 by bisulfite treatment of RNA followed by RT-PCR and Sanger DNA sequencing. In the absence of TbNop2 RNAi, there was 100% methylation of site 1324 (Fig 3A). This is consistent with the high level of methylation observed in the BS-seq dataset. In the presence of TbNop2 RNAi, the level of site 1324 methylation was slightly reduced to 92%, and this trend was also observed in the BS-seq dataset (Fig 3B). Thus, there is not strong evidence that TbNop2 methylates rRNA in *T*. *brucei* under the conditions tested, although we cannot rule out a small contribution.

### rRNA processing in TbNop2 RNAi parasites

**Fig 4:**
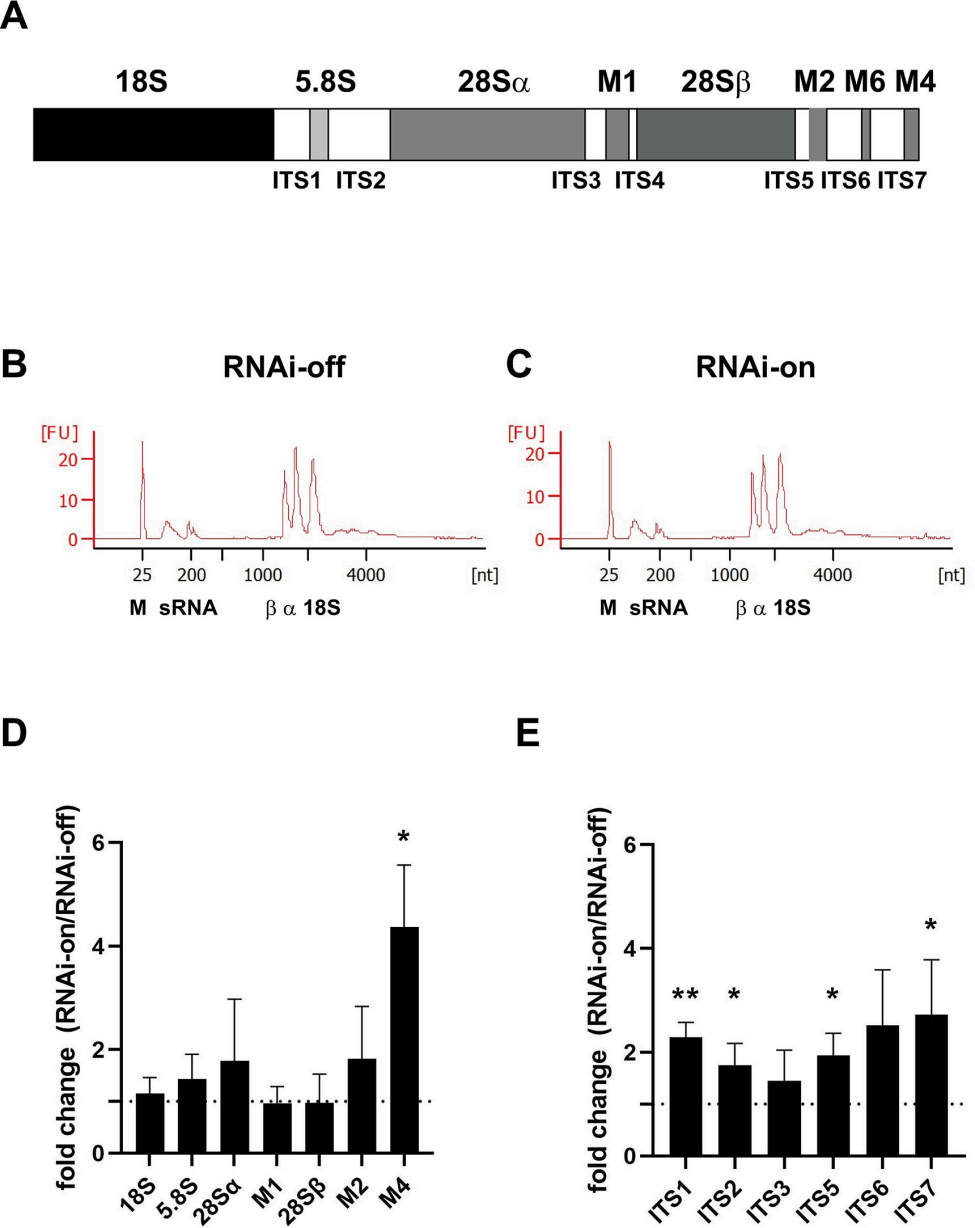
Ribosomal RNA levels and processing in TbNop2 RNAi parasites. A) Ribosomal RNA locus in *T*. *brucei*. The locus is transcribed generating one, long pre-rRNA. The top names refer to rRNA fragments incorporated into the ribosome. The bottom refers to ITS sequences, which are the sites of pre-rRNA cleavage. B) and C) TbNop2-Ty RNAi cells were grown in the absence of doxycycline (B, RNAi-off) and presence of 4 μg/mL doxycycline (C, RNAi-on) for two days. Total RNA was isolated, treated with DNase, and analyzed on an RNA Nano 6000 chip. The X-axis is RNA size in nucleotides, and the Y-axis is fluorescence units. M is the position of the fluorescent marker, sRNA is the size of small RNA including tRNA and small rRNA, α and β indicate the sizes of 28Sα and 28Sβ rRNA, and 18S is the size of 18S rRNA. D) and E) TbNop2-Ty RNAi cells were grown in the absence of doxycycline (RNAi-off) and presence of 4 μg/mL doxycycline (RNAi-on) for two days. Total RNA was isolated and converted to cDNA. cDNA for steady-state rRNAs and ITS sequences were quantified using qPCR. D) represents rRNAs and E) represents ITS sequences. β-tubulin was used as a loading control, and fold-change values were calculated as described in the Materials and Methods (ΔΔCt method). The bars represent average fold-change for each sample and the error bars represent the standard deviation. The dotted vertical line is a fold-change of 1, which represents no difference between RNAi-on cells and RNAi-off cells. Samples were analyzed by one-sample t-tests where * is p<0.05 and ** is p<0.005. The data are from 4 experiments (n = 4, biological replicates).

As Nop2 homologs such as ScNop2 and HsNsun1 impact processing of rRNA, we next examined rRNA levels and rRNA processing in TbNop2-Ty RNAi cells. In *T*. *brucei*, the rRNA locus is transcribed and cleaved into six fragments: 18S rRNA, 28Sα, 28Sβ, M1, M2, M4, and M6 rRNA (Fig 4A). The regions connecting the stable fragments above are ITSs of which there are seven (ITS1- ITS7) (Fig 4A). First, total RNA profiles in TbNop2-Ty RNAi-off and RNAi-on cells were compared using RNA bioanalysis. The profiles in TbNop2-Ty RNAi-off and RNAi-on cells were virtually identical, suggesting the lack of large changes in the levels of abundant, steady-state RNAs such as 18S and 28S rRNA (Fig 4B and 4C). There was no evidence for an increase in large precursor RNAs in the TbNop2-RNAi samples. To test for the presence of small changes in the abundance of specific rRNAs, qRT-PCR was utilized as it is more sensitive than RNA bioanalysis. The steady-state levels of 18S, 28Sα, 28Sβ, M1, M2, and M6 RNAs were primarily unchanged in the presence of TbNop2 RNAi (Fig 4D). The one exception was increased levels of M4 rRNA in the presence of TbNop2 RNAi. We additionally examined 6 of the 7 rRNA ITS regions via qRT-PCR in TbNop2 RNAi cells (Fig 4E). ITS4 was too small to analyze via RT-PCR. Five of the six ITS regions were increased greater than 1.5-fold in the presence of TbNop2 RNAi (the exception was ITS3). Four of the 6 ITSs were increased >1.5-fold with a p-value of <0.05 in one-sample t-tests. Thus, TbNop2 RNAi increases the levels of rRNA precursors. The simplest interpretation is that TbNop2 is required for rRNA processing, and this relationship has been observed for Nop2 family members including ScNop2 [33,34] and HsNsun1 [61].

## Conclusions

Herein, we describe the properties of *T*. *brucei* CRMT homolog TbNop2. TbNop2 is homologous to other CRMTs and has the six highly conserved motifs found in cytosine RNA and DNA methyltransferases, including the two catalytic cysteine residues (Fig 1). When RNAi was used to reduce TbNop2 levels, TbNop2 RNA and protein levels were dramatically reduced (Fig 2A). In addition, TbNop2 RNAi cells stopped dividing after two days of RNAi induction with doxycycline, indicating an important role for TbNop2 in parasite growth (Fig 2B). TbNop2 RNAi cells have increased levels of several rRNA ITS sequences, but do not display changes in the steady-state levels of most mature rRNA species (Fig 4B–4E). Thus, the simplest interpretation of the data is that TbNop2 is also necessary for or enhances rRNA processing. It is certainly possible that the growth defect observed in the TbNop2-Ty RNAi cells is due to a lack of rRNA processing, although it is also possible that TbNop2 has multiple roles in the cell and RNAi-based growth defect is due to some other function.

In *T*. *brucei*, there have been several rRNA processing factors identified including RNA-binding proteins, ribonucleases, small nucleolar RNAs (snoRNAs) and their respect core proteins that direct sugar methylation, base pseudouridylation, and rRNA cleavage [65]. Some of these molecules are highly conserved in other eukaryotes, yet others such as distinct snoRNAs are specific to *T*. *brucei* and close family members. The *T*. *brucei* rRNA processing factors are required for processing of the ~10Kb rRNA precursor into the mature 5.8S, 18S, 28Sα, 28Sβ, and M RNAs via ITS removal. In the TbNop2 RNAi cells, many ITSs are in greater abundance, including the first internal cleavage site ITS1 [65]. This suggests that TbNop2 functions at an early step in pre-rRNA processing and perhaps on the initial pre-rRNA. Thus, TbNop2 shares a rRNA processing role with ScNop2 [22,32–34] and HsNsun1 [61]. Both ScNop2 and HsNsun1 are required for early processing of the large ribosomal RNA; their depletion results in increased levels of the initial, large precursor RNA [22,33,34,61]. ScNop2 is also necessary for conversion of the 27S rRNA to the 25S rRNA, which is downstream from the initial cleavage of the 35S pre-rRNA [33,34]. Yet, our data do not indicate how TbNop2 impacts ITS sequence levels. We do not believe TbNop2 is impacting rRNA processing by directly methylating ITS sequences, as there is little evidence for ITS methylation in our datasets (see S2 Table). However, the impact of TbNop2 could be direct via rRNA precursor binding and/or snoRNA recruitment. For example, HsNsun1 binds to pre-rRNA, multiple snoRNAs, and multiple snoRNA core proteins, and is required for rRNA processing via an methylation-independent pathway [61]. It is certainly worth considering a potential relationship between TbNop2 and *T*. *brucei* snoRNAs such as snoRNA TB11Cs3C2. This *T*. *brucei* snoRNA is required for the early step of ITS1 cleavage, which is reduced in TbNop2 RNAi cells [66]. Alternatively, TbNop2 could have an indirect impact on rRNA processing. For example, TbNop2-RNAi cells may be stressed due to their severe growth defect (Fig 2B), and stress can pause rRNA processing in human cells at early stages in the process [67].

We did not observe a robust loss of cytosine RNA methylation of any rRNA species when TbNop2 was targeted by RNAi (Table 1 and Fig 3), although ScNop2 and HsNsun1 methylate rRNA at sites that are homologous to the *T*. *brucei* site 1324 [22,32,61]. There are several possibilities for this phenomenon. First, it is possible that TbNop2 is a CRMT and methylates rRNA, but there is no loss of RNA methylation in TbNop2 RNAi cells due to the presence of low TbNop2 levels after RNAi (knockdown, not knockout), and/or the presence of another CRMT with overlapping substrate specificity. Of importance, RNA was isolated on day 2 of the RNAi experiment due to severe growth defect. This is the day before the detectable growth defect and was designed purposely to detect early events leading to a cessation of parasite growth. In addition, the observation of a loss of rRNA methylation may take greater than two days as rRNA is known to be extremely stable. Interestingly, only a weak loss of rRNA methylation was observed in studies when ScNop2 was depleted [22]. Second, it is possible that TbNop2 is a methyltransferase, but its target(s) is not rRNA and thus was not identified. We focused specifically rRNAs based on TbNop2 nucleolar location [62] and the relationship to the Nop2 family, yet TbNop2 could methylate RNAs not analyzed in our experiments such as tRNAs. To our knowledge, there is no evidence for cytosine base methylation in *T*. *brucei* mRNA, and thus we did not consider a role for TbNop2 in mRNA cytosine base methylation.

We also detected the presence of six other CRMTs besides TbNop2 (S1 Table). Consequently, it is logical to consider the potential functions for the other *T*. *brucei* CRMT homologs. It is possible that one or more CRMTs are responsible for methylation of tRNA. In *T*. *brucei*, tRNA methylation primarily occurs in the variable region between the anticodon system and T-arm [42]. Many *T*. *brucei* tRNAs have multiple methylated cytosines, and it is possible that multiple enzymes are necessary for complete tRNA modification. We also hypothesize that the other CRMTs are involved in rRNA methylation. Two 28S rRNAβ sites were found in the bisulfite sequencing data without an assignment of the responsible enzymes. As there is no transcriptome-wide methylation map for *T*. *brucei*, it is possible that the CRMTs could methylate other RNA sites. Overall, it is clear that *T*. *brucei* contains an epitranscriptome with several RNA modifications including m^5^C in multiple RNA species including rRNA and tRNA. Future experiments will focus on determining the entire extent of the *T*. *brucei* epitranscriptome, the identification of the responsible enzymes, and the function of the modifications/enzymes.

## Supporting information

S1 Fig Oligonucleotides used for PCR and qPCR. (DOCX)

S2 Fig Original western blot chemiluminescent images used for Fig 2A. (PDF)

S3 Fig *T*. *brucei* rRNA region used for bisulfite sequencing read mapping. (TXT)

S1 Table BLASTP hits from *T*. *brucei* TREU 927 using *Saccharomyces cerevisiae* Trm4 as a query (E value <1E-5). (XLSX)

S2 Table Bisulfite sequencing data of *T*. *brucei* rRNA locus. Data for all cytosines are present. Column A is the rRNA gene or region. Column B (pos) is the position of the cytosine in the sequence. Column C (strand) is strand analyzed. All values are “+” indicating coding strand. Columns with “C” indicate sequences reading as a C (m^5^C). Columns with a “T” indicate sequences reading as a T (non m^5^C). Columns with “CT” indicate the total number of sequencing reads (sum of “C” and “T”). Columns with “%m5C” is the percentage of 5-methylcytosine at a specific position (C/CT)*100. There are two samples each for TbNop2 RNAi-off samples and TbNop2 RNAi-on samples.
(XLSX)
