# Supplementary material for: A role for a Trypanosoma brucei cytosine RNA methyltransferase homolog in ribosomal RNA processing
Source: PLoS One. 2024 Apr 25;19(4):e0298521. doi: 10.1371/journal.pone.0298521 (PMC11045063; doi:10.1371/journal.pone.0298521)
Supplement: S1 Fig — (DOCX) [file pone.0298521.s001.docx]

**S1 Figure. Oligonucleotides used for PCR and qPCR**.

TbNop2-Ty plasmid construction

5’GGGAACTAAAGACCAAGAACAATCGTATCAGTGTCCCGCCGGCCACGAAGAAGTCTCCAAAGAGTAAAGGGAAAAGTGGAGGTTCTGGTAGTGGTTCC3’

5’CCTGACTCAAGACATATCGCTGGGCTTCCACGAACAGGCAAGTAAAAAAGGTTGTATGTGTCCGCATCTCCGTGTTGCGTCCAATTTGAGAGACCTGTGC3’

TbNop2 RNAi plasmid construction (XbaI site and HindIII site underlined)

5'GATCTAGAGTGATGCAGAGGAGCAAGA3'

5'CTAAGCTTCAAGGAAGGAGCAAGTCGAATA3'

qPCR of RNA from TbNop2 RNAi parasites

5.8S rRNA

5'TGGATGACTTGGCTTCCTATTT3'

5'GCCGTTTGCGTTCAAAGATT3'

28Sα rRNA

5'GGTGGTGAACTATGCCTGAA3'

5'TTCGCTCCGATACCCAAATC3'

28Sβ rRNA

5'GGGAAAGAAGACCCTGTTGAG3'

5'CGTTCCGAGTGGTGGTATTT3'

M1 RNA

5'TGTGGAAATGCGAAACACTTG3'

5'CGGAGATGTGGATTATGGTGAA3'

M2 RNA

5'CCTCTGGTTTCTGGAGTTTGT3'

5'TTTCTGGCTTAGAGGCGTTC3'

M4 RNA

5'TCCCTCTCCAAACGAGAGTA3'

5'AATTCCAAGCATCAAGGTTCAG3'

18S rRNA

5’CGGAATGGCACCACAAGAC3’

5’TGGTAAAGTTCCCCGTGTTGA3’

TbNop2

5'CTTCCCTCACGTGCACAATA3'

5'CCTCCTTCTTCAGCCCATTC3'

ITS 1

5'CCGTTTGACATGGGAGATGA3'

5'ACACAGACACTCTAACACACATAC3'

ITS 2

5'CTCCTCGTGTGGTGCATATT3'

5'CATACACCATTGTGCGAGTAGA3'

ITS 3

5'TAGCCATGTGTGTGTGAGTG3'

5'AAGACAGCTCAGTTGTGAAGAG3'

ITS 5

5'CCATCTCAGCACACAACATTTA3'

5'ACACGTGTGTATAACAGCATTA3'

ITS 6

5'TCACTGACGTTGAAGGGAATG3'

5'ACTGGAAGAGACGGAGGTATAA3'

ITS 7

5'GCGGAGGTGTGAGAGGTATTA3'

5'GACGCGCAACAATACAGACA3'

PCR amplification of 28Sβ rRNA after bisulfite treatment (position 1324)

5’TATTATAGGGATAATTGGTTTGTGG3’

5’ATCTCCAACCAAATAAAATAAAACC3’
