# Supplementary figures and images for: A role for a Trypanosoma brucei cytosine RNA methyltransferase homolog in ribosomal RNA processing
Source: PLoS One. 2024 Apr 25;19(4):e0298521. doi: 10.1371/journal.pone.0298521 (PMC11045063; doi:10.1371/journal.pone.0298521)

**S2 Fig. Original chemiluminescence western blot images used to create Fig 2A.**

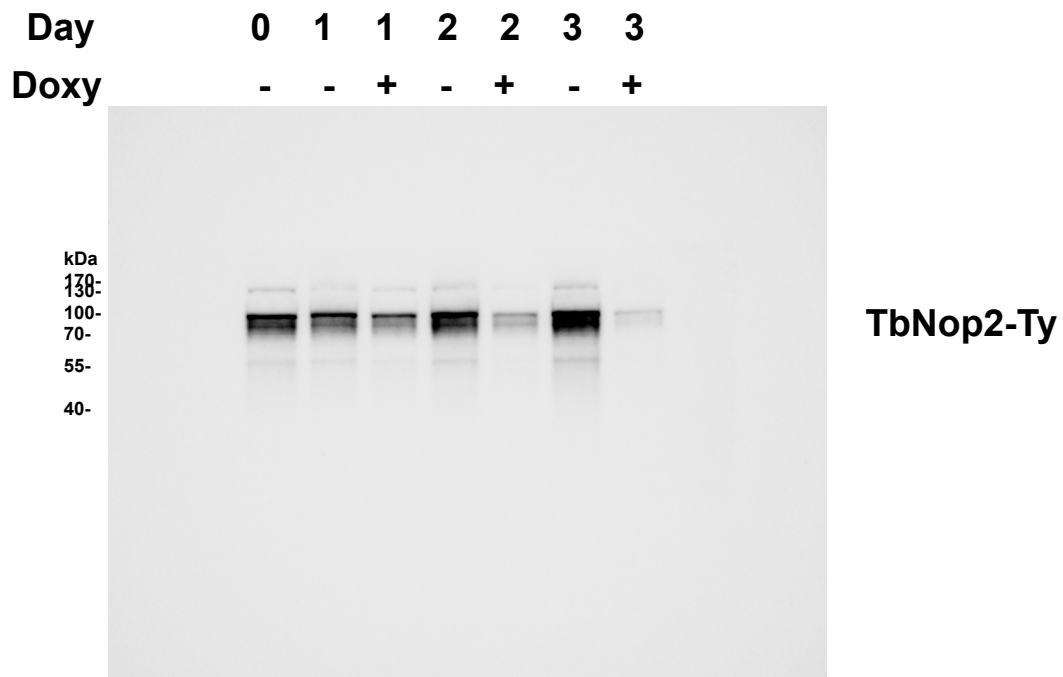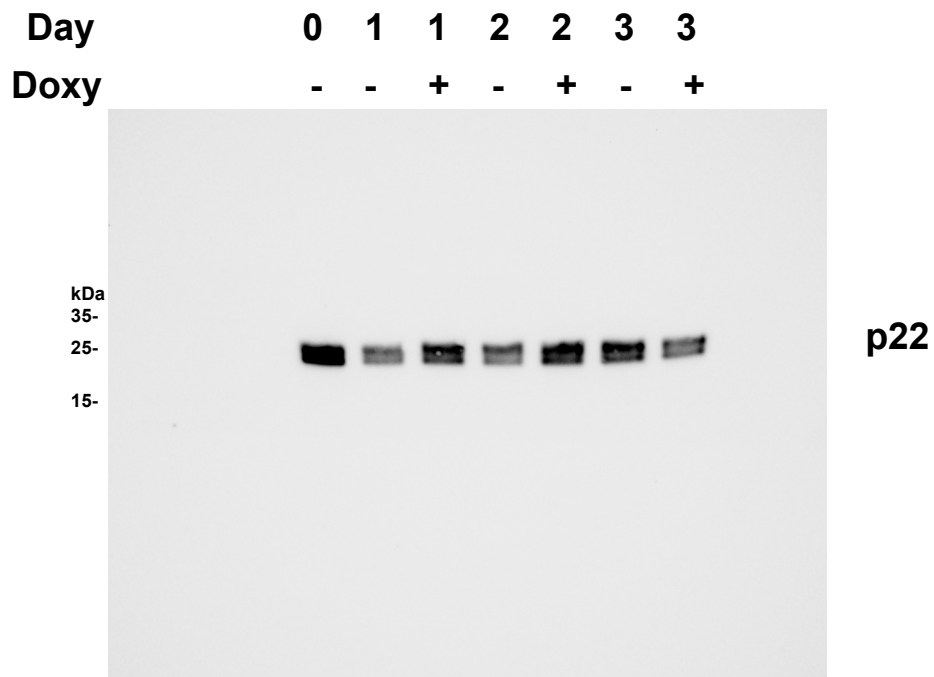

Supplement: S2 Fig — (PDF) [file pone.0298521.s002.pdf]
